# Risk and asset-based strategies in health: priorities in biomedical, life and environmental science literature since the early twentieth century. A rapid review

**DOI:** 10.1186/s12940-022-00833-3

**Published:** 2022-01-29

**Authors:** Virginie Migeot, Jérémy Guihenneuc, Houria El Ouazzani, Marion Albouy, Antoine Dupuis, Sylvie Rabouan

**Affiliations:** 1 School of Medicine and Pharmacy, University of Poitiers, 6 rue de la Milétrie, TSA 51115, 86073 Poitiers Cedex, France; 2 Clinical Investigation Center, INSERM, 2 rue de la Milétrie 1402, 86021 Poitiers, France; 3 Biology-Pharmacy-Public Health Department, University Hospital of Poitiers, 2 rue de la Milétrie, 86021 Poitiers, France; 4 University of Poitiers, CNRS UMR7267, Ecologie & Biologie des Interactions, 86000 Poitiers, France

**Keywords:** Review, Risk factors, Scientific literature, Health assets, Resilience, COVID-19, One Health

## Abstract

**Background:**

In biomedical, life or environmental science research, two different strategies exist depending on the starting point of the researchers: “what makes us ill? “ or “what makes us healthy?”. Indeed, a risk-based strategy (RBS) attempts to minimize risk factors increasing the likelihood of developing a disease, while an asset-based strategy (ABS) attempts to promote and strengthen the factors that support good health and wellbeing. We provided an up-to-date overview of both research strategies in peer-reviewed scientific literature, in the fields of human health, animal and plant health and ecosystem health, to fit with the One Health framework. More particularly, we focused on human health by studying publications related to the COVID-19 at the beginning of the pandemic.

**Design:**

A rapid review of research science literature was carried out to identify in the PubMed/MEDLINE database the proportion of peer-reviewed articles adopting either a RBS or an ABS, in the main global environment fields from January 01, 1900 to December 31, 2019 and, related to COVID-19, from December 1, 2019 to May 31, 2020.

**Results:**

The number of published articles resulting from our search was 1,957,905, including 91.3% with an RBS and 8.7% with an ABS. When examining each field, we found that only 10.5% of human health articles deal with ABS, 5.5% for animal health, 2.2% for ecosystem health, 1.0% for plant health and 2.7% for environmental media. We noted that articles adopting both strategies were published in all health fields. Among the articles concerning COVID-19, 5,854 (55.9%), 542 articles (5.2%) adopted RBS and ABS, respectively, while 4069 (38.9%) simultaneously presenting both strategies.

**Conclusion:**

Our results have allowed us to take stock of the biomedical research strategies prioritized during the twentieth century. It seems highly likely that the two strategies we have analyzed can now be chosen in such a way as to promote a balance in public health measures, at every level to guide One Health interventions aimed at helping people, animals, and plants to lead healthier lives.

## Background

Throughout history, epidemics have accompanied humanity [1–4]. Notwithstanding the medical progress achieved over the nineteenth and twentieth centuries (hygienic advances, the discovery of antibiotics), they led many to think that humanity was on the verge of attenuating if not eliminating the scourges of infectious disease [5]. The early twenty-first century has been marked by successive epidemics, of which the most recent, COVID-19, is by no means under complete control. The coronavirus brings into focus the three following major elements: 1- Globalized sanitary risk [6], which appears as an upshot as much of globalization itself (from 1980 to 2017, the volume of world trade was multiplied by 6.8, while global GNP was multiplied by 3.5 [7], as of economic, political and medical regulation on a worldwide scale by a plethora of organizations including the World Trade Organization, the International Monetary Fund, the International Labor Organization, the World Health Organization, etc.… [8–10]; 2- The chronic disease burden [11–14] was foregrounded in 2005 by a World Health Organization report entitled “Preventing Chronic Diseases: a vital investment”, given that chronic diseases accounted for 60% of deaths throughout the world [15]. As regards COVID-19, in a 21 May 2020 epidemiological assessment [16], *Santé Publique France* observed that 86% of coronavirus-related deaths involved comorbidities, and on 30 May 2020 [17], the USA Center of Disease Control reported 6 times more hospitalizations and 12 times more deaths for COVID-19 patients with underlying conditions, of which the most frequent were cardiovascular disease (32%), diabetes (30%) and chronic lung disease (18%). To conclude, as R.J. Jackson declared in 2007, the seemingly insurmountable health challenge of the twenty-first century would consist in “a mix of global warming, poverty, and infectious and chronic diseases” [5]. 3- The link between health and environment [5, 18] and the imperious necessity of creating a world in which humans would be attuned to more than humankind alone [18]. With that in mind, societies are called upon to transform themselves [19] and to understand that humans, animals and the natural environment are inextricably interconnected [20]. This has proved propitious to several concepts developed at the outset of the twenty-first century: (i) “One Health, One World” was initiated in 2004 by the World Conservation Society and pursued in 2008 under the term “One Health”, the initial objective being to control emerging zoonotic viruses; nowadays, it is more broadly dedicated to public health among humans and to animal health with its repercussions on human health, as epitomized by ecosystems [21–24]; (ii) “Ecohealth” likewise emerged in 2004 as a new research field addressing the complexly interwoven relationships among humans, animals, and the environment, and their impact on health in each domain [20, 25]; (iii) “Planetary Health” was created in 2015 with the objective of transforming public health by taking into account the ecosystems surrounding populations, and it has called for a social movement to support collective public health at all levels of society—personal, community, national, regional, global, and planetary [23, 26]. Although these different concepts have different histories, One Health Global Network considered that their core message is similar and called for a “whole of society” approach to improve health at an optimal level. This widespread approach includes not only plant health, soil health, agricultural systems but also well-being, social and cultural drivers, perception of health and benefits of nature to human health [27]. As for Lerner et al., he explained that one of the most obvious differences between these concepts consists in their views on health and advocated further philosophical concept analysis to develop definitions of health suitable to a merged approach [20]. Whereas One Health is still mainly oriented towards preventive medicine, probably because of its roots in One Medicine [28], It presently appears that the time-worn risk-based strategy no longer suffices. Perhaps, the COVID-19 pandemic has shed light on the possible shortcomings of research conducive to a vision limited to a risk-based strategy (RBS), which identifies and attempts to minimize risk factors [29] increasing the likelihood of developing a disease or suffering from a trauma. By contrast, action may also be based on the asset-based strategy (ABS), which identifies resources that could enhance the ability of individuals, groups, communities, populations, social systems and /or institutions to maintain and sustain health and wellbeing and to reduce health inequalities [30, 31]. In other words, it appears possible to identify health assets from among the health determinants [32] corresponding to the personal, social, economic and environmental factors influencing the health status of persons and populations alike. On that score, where do we stand at present?

In order to address this question from a health-related standpoint, we propose as a first step to acquire more in-depth understanding of scientific research regarding the two strategies (risk-based and asset-based) in the widespread One Health approach including human health, animal and plant health as well as ecosystem health and their interfaces, with regard to seven layers: soils, water, air, plants, animals, ecosystems, humans [33–35].

As a second step, we will put the comparative approach into practice, shedding light on human health alone by providing an overview of publications having to do with the COVID-19 pandemic.

## Methods

We have undertaken a rapid review of biomedical and life science research articles to identify the proportion of articles adopting either RBS or ABS pertaining to the main domains in the global environment.

A rapid review can be defined as a form of knowledge synthesis in which components of the systematic review process known as “evidence summaries” are streamlined to produce information in a short period of time [36]. Rapid reviews can be particularly useful by providing timely evidence to inform decision-making or produce guidance for health systems [37]. Although numerous rapid reviews have been produced, their varied methodologies can diversely impact research results [38–40].

### Protocol

A rapid review protocol was compiled based on the ‘framework of rapid review methods’ by Tricco et al. and from the World Health Organization practical guide [38, 41]. Hence, as we adopted a bibliometric approach to conduct this review, we did not directly involve any patient or any other kind of population in this research.

### Information sources and literature search

In our search in PubMed® for relevant literature, we limited ourselves to the main fields of the global environment in texts published from January 1, 1900 to December 31, 2019 and from December 1, 2019 to May 31, 2020 in those regarding COVID-19. No exclusion criteria other than the date were applied. Articles in all languages could be included. If we chose to focus on PubMed®, it was because it provides i) comprehensive coverage of research across multiple disciplines of life sciences, and ii) a controlled vocabulary system and standardized indexation of articles by the National Library of Medicine with a Medical Subject Headings (MeSH) thesaurus [42]. Our choice allowed us to carry out a precise, defined, relevant and representative search. Wishing to identify the most relevant terms designating the seven fields (soils, water, air, plants, animals, ecosystems, humans: Table 1), we included only MeSH terms or specific terms mentioned in article titles, our objective being to avoid excessive “noise” in our research results. In case of hesitation between two MeSH terms, we chose the one closest to the root of the tree. Search in the “grey” literature for additional articles was not conducted.

**Table 1:** Relevant terms for each field of research, PubMed® and signification of MeSH terms

Areas of research	PubMed® equation	Signification of MeSH term
Soils	Soil[MeSH]	Soils, clay, humic substances, permafrost, sand, peat, humus
Water	Water[MeSH] OR “Water Resources”[MeSH] OR Groundwater[Mesh] OR “Fresh Water”[Mesh] OR “Saline Waters”[Mesh]	Water, hydrogen oxide, deuterium oxide, drinking water, ice, mineral water, steam, water resources, groundwater, aquifers, lakes, ponds, rivers, seawater, oceans, seas
Air	Atmosphere[MeSH]	Air, weather, climate, atmospheric pressure, stratospheric ozone
Plants	Plants[MeSH] OR “green space”[Title] OR “green spaces”[Title]	Crops, glaucophyla, plant weeds, seedlings, trees, viridiplantae, green spaces
Animals	Animals[MeSH] NOT Humans[MeSH]	Animals, animal population groups, chordata, invertebrates
Ecosystems	Ecosystem[MeSH]	Biodiversity, biota, biomass, coral reefs, food chain, forests, rhizosphere
Humans	Humans[MeSH]	Humans

### Selection and screening process

We identified the most relevant terms designating a disease-oriented strategy and an RBS. Keywords were sought out in several English-language reference medical dictionaries by a reviewer (JG) and selected by the team members (HEO, VM) once a consensus had been reached, subsequent to a series of pilot tests. The terms we selected were sufficiently precise to avoid off-topic research. The selection process and final equation are detailed in Table 2. Relevant MeSH terms such as “disease” or “health risk behaviors” were included. We excluded i) terms for which the results were largely irrelevant (“cause”, “damage” or “etiology”), ii) terms included in relevant MeSH terms (“exposure”), iii) MeSH terms representing a subset of another MeSH term closer to the root of the MeSH tree (“environmental exposure”) and iv) terms for which the definition of MeSH or research results did not allow for their classification as a RBS (“social determinants of health)”. Since the MeSH term "epidemiologic factors" encompasses more notions than “protective factors” and “risk factors” alone, it was excluded.

**Table 2:**
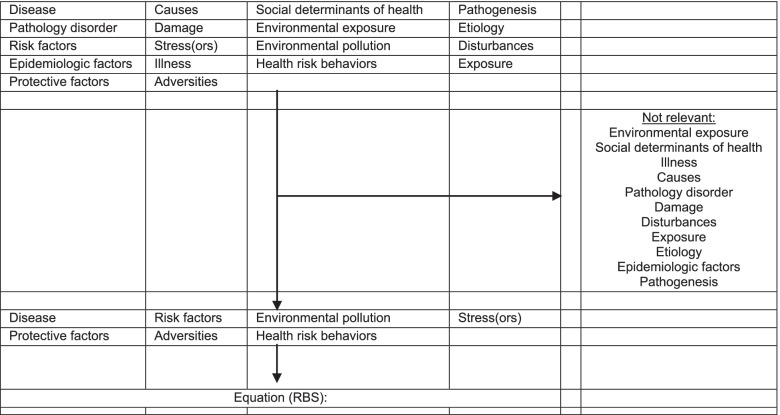
Selection process and final equation (RBS) of the most relevant keywords in risk-based strategy, PubMed®

"Risk factors"[Mesh] OR “protective factors”[MeSH Terms] OR “Disease”[Mesh] OR stress*[Title] OR "health risk behaviors"[Mesh] OR "Environmental pollution"[Mesh] OR adversit*[Title]

We used the same selection process to identify the most relevant keywords designating a health-oriented strategy aimed at obtaining the final ABS equation (Table 3). We included relevant terms (“resilience”, “adaptation”) and excluded i) terms for which results were largely irrelevant (“humor”, “interdisciplinarity”, ii) terms included in MeSH terms (“coping”), iii) MeSH terms representing subsets of another MeSH term closer to the root of the MeSH tree (“post-traumatic personal growth”), iv) terms for which MeSH definition or research results did not allow for their classification as a health resources approach (“health”) and v) terms for which there were no results (“will to meaning”).

**Table 3:**
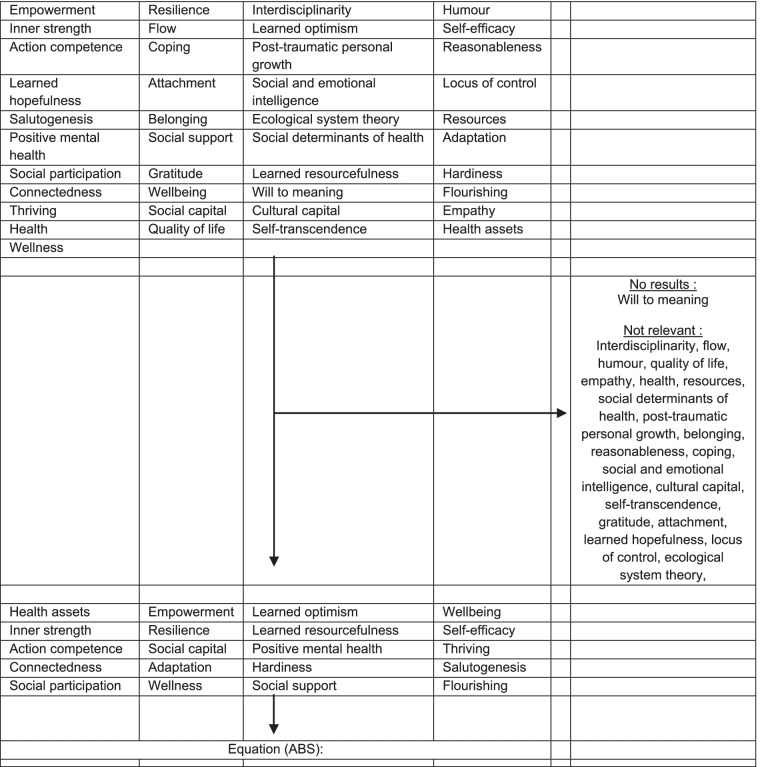
Selection process and final equation (ABS) of the most relevant keywords in health-oriented strategy, PubMed®

“Adaptation, Psychological”[Mesh] OR “Resilience, Psychological”[Mesh] OR resilien*[Title] OR "health asset"[Title] OR "health assets"[Title] OR salutogen*[Title] OR empower*[Title] OR “positive mental health”[Title] OR “social participation”[Title] OR “action competence”[Title] OR “hardiness”[Title] OR “connectedness”[Title] OR “inner strength”[Title] OR “learned optimism”[Title] OR “self-efficacy”[Title] OR flourishing[Title] OR thriving[Title] OR “well-being”[Title] OR “wellbeing”[Title] OR “wellness”[Title] OR “social capital”[Title] OR “learned resourcefulness”[Title] OR “social support”[Title]

The same approach as above was applied to the COVID-19 disease. A few changes were carried out (search period, keywords).

On this subject, we searched in PubMed® from December 1, 2019 to May 31, 2020. Delays in indexing MeSH terms to published articles made it impossible to perform a search based solely on these terms. Using a multilingual health terminology portal [43], we constructed an equation enabling us to obtain a complete list of articles published with respect to COVID-19: "COVID-19"[Supplementary Concept] OR "2019 novel coronavirus disease"[Title/Abstract] OR "2019 novel coronavirus infection"[Title/Abstract] OR "2019-nCoV disease"[Title/Abstract] OR "2019-nCoV infection"[Title/Abstract] OR "coronavirus disease 2019"[Title/Abstract] OR "coronavirus disease-19"[Title/Abstract] OR "COVID19"[Title/Abstract] OR "COVID-19 pandemic"[Title/Abstract] OR "COVID-19 virus disease"[Title/Abstract] OR "COVID-19 virus infection"[Title/Abstract] OR "SARS-CoV-2 infection"[Title/Abstract] OR "severe acute respiratory syndrome coronavirus 2"[Supplementary Concept] OR "severe acute respiratory syndrome coronavirus 2"[Title/Abstract] OR "2019 novel coronavirus"[Title/Abstract] OR "2019-nCoV"[Title/Abstract] OR "coronavirus disease 2019 virus"[Title/Abstract] OR "COVID19 virus" [Title/Abstract] OR "SARS-CoV-2" [Title/Abstract].

Then, starting from previous equations [(RBS) and (ABS)], we replaced MeSH terms with another “entry term” in PubMed® to maintain to the greatest possible extent the same research field and equation structure. Regarding the RBS equation, we explored PubMed® with the following keywords: “risk factor*”, “protective factor*”, “risk*”, “illness”, “stress*”, “health risk behavior*”, “pollution”, “adversity*”. For the ABS equation, the following keywords were included: “adaptation”, “coping”, “resilience*”, "health asset*", “salutogen*”, “empower*”, “positive mental health”, “social participation”, “action competence”, “hardiness”, “connectedness”, “inner strength”, “learned optimism”, “self-efficacy”, “flourishing”, “thriving”, “well-being”, “wellbeing”, “wellness”, “social capital”, “learned resourcefulness” and “social support”.

To determine which articles specifically discussed only a single strategy, we excluded the opposing equation by using the Boolean operator NOT.

### Quality appraisal

Two reviewers (JG, VM) independently took a random sample of articles (*n* = 30) from each field and each strategy to ensure correct classification. A third reviewer (HEO) mediated when consensus was not reached.

### Statistical analysis

The proportion of published research articles adopting an ABS was compared to those adopting an RBS in several fields. As an example, comparison in human health about the current COVID-19 crisis was carried out.

Comparisons of percentages were calculated using the percentage comparison z-test. A two-sided alpha level of 0.05 was chosen for statistical significance for all analyses.

## Results

The number of articles resulting from equations (RBS) and (ABS) of the PubMed® research is as follows: the RBS equation (RBS), led to 1,957,905 (91.3%) results while 185,726 articles resulted from the ABS equation (ABS). Articles on health using an ABS were significantly fewer (*p*-value < 0.001) and represented 8.7% of all articles. Results are presented in Table 4. For all the fields, each strategy could be identified through the search equations: from 89.5% to 99.7% for the RBS and from 0.3% to 10.5% for the ABS.

**Table 4:** Article distribution in the different fields according to the strategies used (ABS or RBS) between January 01, 1900 and December 31, 2019 (data collected in PubMed®)

	Total	Proportion of each field	Risk-based strategy (RBS)		Asset-based strategy (ABS)		*p*-value
	n	%	n	%	n	%	
All fields	2,143,631	100.0	1,957,905	91.3	185,726	8.7	< 0.001
Humans	1,623,910	75.8	1,453,184	89.5	170,726	10.5	< 0.001
Animals	198,623	9.3	187,616	94.5	11,007	5.5	< 0.001
Plants	89,416	4.2	88,507	99.0	909	1.0	< 0.001
Ecosystems	45,326	2.1	44,350	97.8	976	2.2	< 0.001
Air	93,341	4.3	91,796	98.3	1545	1.7	< 0.001
Water	67,249	3.1	66,767	99.3	482	0.7	< 0.001
Soils	25,766	1.2	25,685	99.7	81	0.3	< 0.001

Concerning the period between January 1, 1900 and December 31, 2019, 1,623,910 articles on human health were included. An RBS was chosen in 1,453,184 (89.5%), and an ABS in 170,726 articles (10.5%). Regarding ecosystem health, 45,326 articles were included, and 44,350 articles (97.8%) adopted an RBS, while only 976 articles (2.2%) adopted an ABS. Few articles on the ABS were found in animal health, with only 11,007 (5.5%) among 198,623 articles regarding this topic. The ABS was even less present in the field of plant health with 909 articles (1.0%) and in health of environmental media with 1545 articles (1.7%) on air, 482 articles (0.7%) on water and 81 articles on soil (0.3%). In each field, the number of published articles presenting an RBS was significantly higher (*p*-value < 0.001) than those adopting an ABS.

**Fig. 1:**
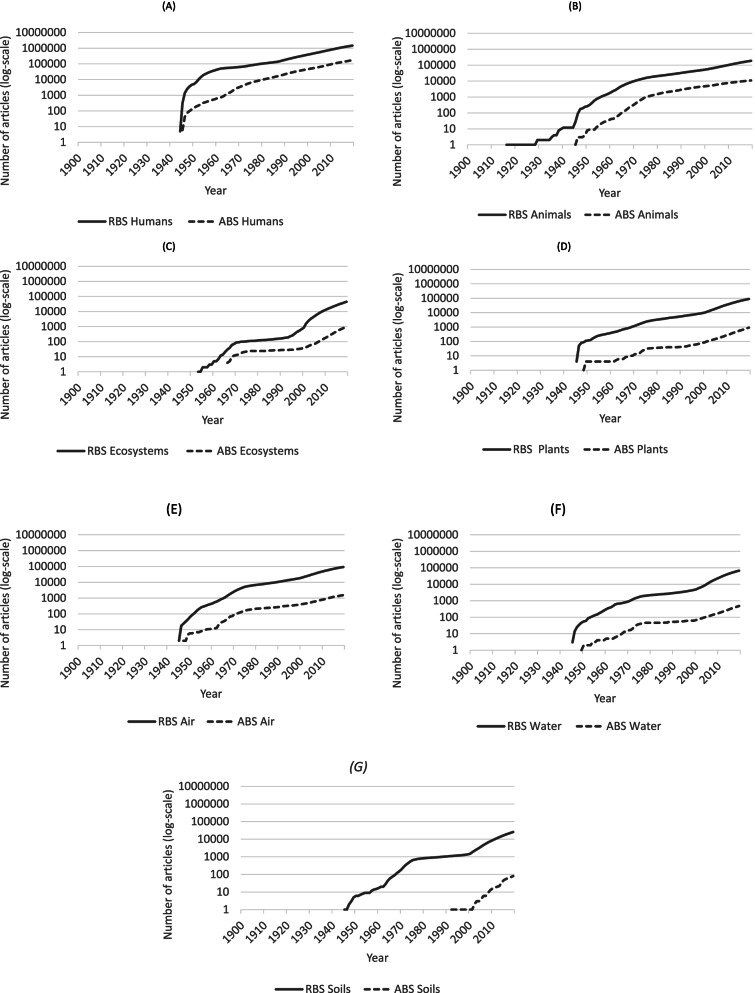
Cumulative number in logarithmic scale of PubMed® published articles according to the different fields adopting asset-based strategy (ABS) or risk- based strategy (RBS), between January 01, 1900 and December 31, 2019 (data collected in PubMed®)

It bears mentioning that articles adopting both strategies were published in all health fields: 17,241 (1.1%) in human health, 146 (0.3%) in ecosystem health, 799 (0.4%) in animal health, 40 (0.0%) in plant health, and 258 articles in health of environmental media, with 179 (0.2%) on air, 63 (0.1%) on water and 16 (0.1%) on soil. Figure 1 shows that in most fields, articles adopting an RBS have been referenced since the 1940s. In ecosystem health, articles began later than in the other fields (Fig. 1C). In animal health (Fig. 1B), we have numbered 12 articles, including 10 in the same journal before 1940 (data not shown).

Articles on Ecosystem health have been published later than the other fields, in 1953 for the articles adopting an RBS and in 1966 for those with ABS (Fig. 1C).

Articles presenting an ABS have been published as the same time as those with RBS in all seven fields, except for soil health, which started only in the 1990’s (Fig. 1D).

**Fig. 2:**
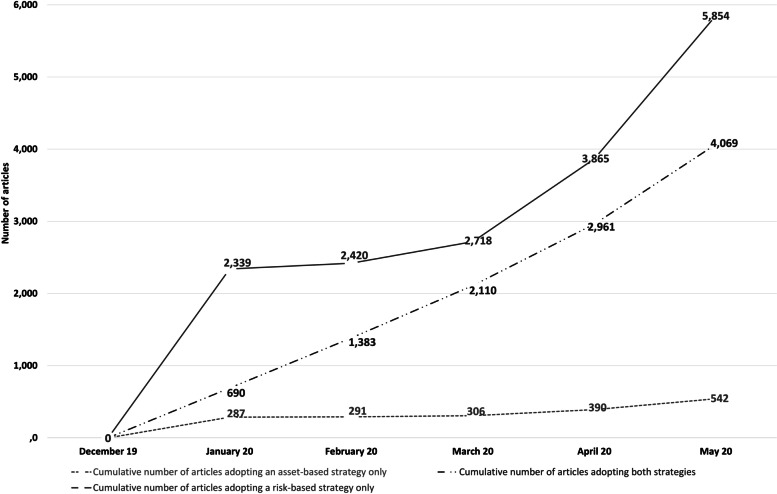
Cumulative number of PubMed® published articles on COVID-19 adopting an asset-based strategy (ABS), a risk- based strategy (RBS) or both strategies (from December 2019 to May 2020)

Taking as an example the COVID-19 disease, among the 25,642 articles published with respect to COVID-19, 10,465 articles (40.8%) were included and classified in an RBS, an ABS or in the adoption of both strategies (Fig. 2).

Among the articles included, the first were published in January 2020 with 2,339 (70.5%) adopting an RBS and 287 articles (8.7%) an ABS. Up until May 31, 2020, the cumulative number of published articles was 5,854 (55.9%) and 542 articles (5.2%) adopted an RBS and an ABS, respectively.

In January 2020, 690 articles (20.8%) adopting both strategies were published. The number of these articles increased, and in May, they numbered 4,069 (38.9%).

## Discussion

This rapid review fitted into the framework of the new integrative concepts such as One Health, which recognizes the interconnectedness of people, animal and ecosystems health [48]. Hence, since 2004, One Health and Ecohealth programs have been studying health by focusing on the human-animal-ecosystem interface [44]. In our study, a larger number of research fields in the life and biomedical sciences were included than in previous works [44, 45]. We have considered not only human health or animal health, but also and simultaneously human health, animal health, ecosystem health, plant health, and health of environmental media (soil, water and air). If it bears mentioning that the concept of health was originally formulated as regards the human or animal population [45], it is also used as a metaphor for the ecosystem [20].

Results of this research have been presented throughout synthetic graphics allowing visual overview since the early twentieth century. We showed that in most fields, articles adopting RBS have been referenced since the 1940s, coinciding with the beginning of PubMed®. Comparatively with the other fields, we found that articles on Ecosystem health have been published later than on the other fields. This delay may be explained by the fact that this concept was first used by Arthur George Tansley only in 1935 [46]. This English botanist was already aware of the interconnectedness of the systems and drew attention to the importance to not separate organisms from their environments, with which they form “one system”. On this subject, Van Bruggen et al*.* also argue that the health conditions of all organisms in an ecosystem are interconnected through the cycling of subsets of microbial communities from the environment (in particular the soil) to plants, animals and humans, and back into the environment [47].

To our knowledge, our review is the first to present both RBS and ABS strategies for the seven fields. It highlighted that since the early twentieth century, peer-reviewed scientific articles adopting an RBS accounted for 91.3% of all those published in the biomedical, life, and environmental scientific literature. In 1995, Skolbekken already had shown the increasing trend of the term “risk” in the medical literature from 1967 to 1991. He pointed out that this “risk epidemic” could not be explained as a change in terminology alone, but rather as a result of developments in science and technology that had shifted beliefs about the locus of control from “factors outside human control” to “factors inside human control” [48]. Firstly, this impressive result could be explained, according to Morabia, by the emergence of epidemiology and of the concepts of “exposure” and “outcome”. Health research which was and is designed to investigate the role of health determinants that epidemiologists still study today seems to be more focused on "one exposure" and "one outcome", thereby shifting from a “holistic” to a “reductionist” approach [49]. A disease was defined by the presence or absence of measurable biophysical indicators of disease (and, in some cases, at risks), which were cumulatively defined as standards [50, 51].

Moreover, we can hypothesize that the disproportionately high share of published research articles adopting RBS as opposed to ABS could be largely explained by the Matthew effect in science: “the rich become richer”, “the risk-strategy begets the risk-strategy” [52]. Indeed, the journal in which papers are published has a pronounced influence on their citation rates, Lariviere showed a specific Matthew effect attached to journals that over and above their intrinsic quality endows published papers with an added value [53]. Moreover, Bol et al.investigated to extent to which the Matthew effect determines the allocation of research funds [54]. We could assume that early funding for RBS papers was an asset contributing to acquisition of later funding with the same strategy, at the expense of ABS.

Secondly, the high proportion of RBS-based published research could be explained by a health vision mitigating the importance of the continuum Health-Disease or ease/disease and positioning the slider of health status on risk as opposed to resources [55]. Hence, several authors have highlighted the importance of counterbalancing the "deficit models" [30, 31, 56], which are focused on identifying health problems, illness and health-risk behaviors, through emphasis on positive, asset-based models. As previously mentioned, health assets can appear in an individual, group, community, and /or population as protective (or promoting) factors to “buffer against life’s stresses” [30]. In numerous studies on the resources key concepts have been identified: resilience, wellbeing, self-efficacy, salutogenesis [57], which is why, in the ABS equation, we included more than twenty keywords covering these concepts. Initially defined in the field of ecology, resilience has shifted towards human ecology (i.e. social sciences) [58, 59]. As for salutogenesis, it is an umbrella concept focusing on origins of health rather than origins of disease and encompassing a number of positive approaches [57]. The salutogenic model of health suggests that each individual disposes of various health-creating resources that are diversely acquired (socio-cultural and historical context as well as child-rearing patterns or chance) [60].

Although ABS articles were statistically fewer than RBS articles, we found articles adopting an ABS for all the seven fields: from 81 (0.3%) articles for the soil to 170,726 (10.5%) for humans. For example, this ABS was proposed by Döring et al., who examined the health of the whole planet through the same lens, using the criterion of resilience [65]. In our review, we observed that articles on the health of the soil adopting an ABS were published later than on the other fields. The concept of living soil had been forgotten for many years and then again reconsidered as a living system of which the quality results from multiple interactions among physicochemical and biological components, notably microbial communities, which are primordial for soil function [35, 61].

Regarding research on the COVID-19 pandemic, we observed that during the first months of the outbreak, the risk-based strategy remained predominant (55.9% with RBS vs 5,2% with ABS), but 4,069 (38.9%) articles simultaneously presenting both strategies were published. Throughout the world almost all governments took unprecedentedly drastic measures, suspending virtually all economic, cultural, and social activities. In many countries, complete lock-down was aimed at controlling the spread of the coronavirus and flattening the epidemic peak. The population learned to adopt “barrier gestures” while different fear and risk-based communication strategies were implemented to improve health literacy [62] and foster behavior changes [63]. In accordance with a logic of cure and risk, Van den Broucke stated: “The real war heroes in the battle against the CoV-2 virus are virologists, epidemiologists, doctors and nurses, and even if many of the actions taken serve a preventative purpose, their focus is on the prevention of disease, not on promoting health” [64]. Indeed, to deal with a previously unknown disease and guide medical teams around the world, published information on the virus, its clinical characteristics and the diagnostic and therapeutic strategies tested predominated. To facilitate the dissemination of research results, since the beginning of the COVID-19 outbreak the National Library of Medicine website https://www.nih.gov/coronavirus has been publishing daily updates, while medical scientists have been sharing their findings online prior to peer review by preprint papers [65]. Since March 2020, in published research on the relevance of Ecohealth, One Health and Planetary Health frameworks in the pandemic context generated by COVID-19 [24, 66], some resource-based articles underlining an urgent need to go beyond protective measures against the virus have been published. To prevent and mitigate pathogen emergence and transmission, Roche et al. advocated for “ecosystem management” including restoration, rewilding and the management of wildlife reserves and called on scientists to develop “solution-oriented” research [66]. Most of the other published articles have focused on the need to increase individual and social resilience and to deploy strategies aimed at developing a salutogenic society, as advocated by international public health associations such as EUPHA and IUHPE [67].

In this period, while many countries remain preoccupied by the successive waves of COVID-19, it seems particularly urgent to think about the relevant public health strategies to be developed. Our results have allowed us to take stock of the biomedical research strategies prioritized during the twentieth century. It seems highly likely that the two strategies we have analyzed can now be chosen in such a way as to promote a balance in public health measures, at every level: the individual [68], the city [69], the hospital [70], the health system [71] and even the country [72] as well as the planet [26].

To guarantee the rigor of the study, we constructed several search equations on the PubMed® database using MeSH terms. This strategy enabled an accurate, relevant and representative search in all languages through a controlled vocabulary system and standardized indexation [42].

However, our search strategy based on the PubMed® database limited us to relevant literature on life and biomedical sciences [73]. We did not consider the Web of Science database, which is nonetheless largely integrated in the PubMed® database. Another limitation of the study was the need to rapidly choose between different types of review in order to address our primary objective with illustrative and representative research [74]. Finally, the material studied in these different fields and over this period represented more than 1.8 million published articles. In keeping with our approach, we did not systematically read the articles found in our search in view of verifying their classification between or among the two strategies. However, following a random check on the results, our classification seems to be appropriate and accurate. Publications simultaneously addressing the two strategies represented only 0 to 1.1% of all articles.

As demonstrated above, the study of a single health field cannot be fully relevant without considering the others; human health must not be studied separately from the global environment. The Sars-CoV-2 example illustrates the need to take all relevant fields into account in view of preserving human health by developing five key principles of health promotion: intersectorality, sustainability, empowerment and public engagement, equity and a life course perspective [67].

## Conclusion

During the twentieth century and even now, health research has focused on RBS, and the ABS approach has been given too little emphasis. More studies must be conducted to promote the ABS approach in all health fields, and financial support needs to be provided to redress the balance between the two strategies. The concepts of pathogenesis and salutogenesis in scientific research are to be considered complementarily. Improved understanding of health could guide interventions aimed at helping people, animals, and plants to lead healthier lives.

## Data Availability

The datasets used and/or analysed during the current study are available from the corresponding author on reasonable request.

## Abbreviations

ABS: Asset-Based Strategy
MeSH: Medical Subject Headings
RBS: Risk-Based Strategy
